# Association Between the Dietary Index for Gut Microbiota and Metabolic Syndrome: Mediation Effects of Albumin and Systemic Immune‐Inflammation Index

**DOI:** 10.1002/fsn3.71194

**Published:** 2025-11-11

**Authors:** Shouxin Wei, Sijia Yu, Chuan Qian

**Affiliations:** ^1^ Department of Gastrointestinal Surgery Suining Central Hospital Suining China; ^2^ Department of General Practice Suining Central Hospital Suining China

**Keywords:** cross‐sectional study, dietary index for gut microbiota, gut microbiota, metabolic syndrome, NHANES

## Abstract

With the development of society, the prevalence of metabolic syndrome (MetS) has been increasing year by year and has become a major global health threat. The dietary index for gut microbiota (DI‐GM), a recently proposed dietary assessment metric, has attracted considerable attention. However, its association with MetS has lacked systematic research evidence. This study was based on data from 59,842 nationally representative participants in the 2007–2018 National Health and Nutrition Examination Survey (NHANES) database, using methods such as multivariable weighted logistic regression, restricted cubic splines (RCS), subgroup analysis, and mediation effect analysis to explore the association between DI‐GM and MetS. The study showed a significant negative correlation between the DI‐GM score and the risk of MetS (OR = 0.947 [0.921, 0.974]), and as the DI‐GM score increases, this association becomes more pronounced. Subgroup analysis found no significant interactions, while mediation effect analysis further revealed that serum albumin and the systemic immune–inflammation index (SII) played a partial mediating role in this association. This study is the first to confirm a significant negative correlation between DI‐GM and MetS risk, suggesting that optimizing dietary structure may be a feasible intervention for the prevention and treatment of metabolic syndrome.

## Graphical abstract

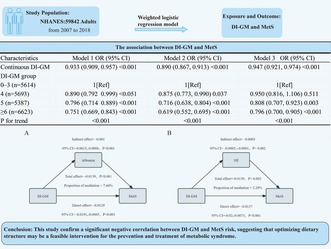

## 1 Introduction

Metabolic syndrome (MetS) is a complex metabolic disorder typically characterized by a clustering of various clinical conditions, including abdominal obesity, hypertension, dysglycemia, and dyslipidemia (Rothwell et al. 2022). Since the World Health Organization (WHO) conceptualized it as the “X syndrome” in 1988, MetS has been recognized as a global health challenge, affecting approximately 25% of the adult population (Corbi‐Cobo‐Losey et al. 2023). With the progression of urbanization and dietary changes, its prevalence is rising annually, particularly in developing countries (Park and Liu 2023). Epidemiological studies have shown that metabolic syndrome not only increases the risk of cardiovascular diseases, non‐alcoholic fatty liver disease, and type 2 diabetes but is also closely associated with the onset of various cancers, making it a significant threat to human health (Chen et al. 2025; Wu et al. 2024).

The gut microbiota refers to a community of microorganisms, including bacteria, fungi, and viruses, that inhabit the human gut, with an estimated total of up to one trillion species. These microorganisms play a crucial role in the host's metabolism, immune system, and overall health (Gomes et al. 2018; Chen et al. 2024). Through their metabolic activities, gut microbiota produce various important metabolic products, the most well‐known of which are short‐chain fatty acids (SCFAs), such as acetate, propionate, and butyrate. These compounds are produced by intestinal bacteria through the fermentation of dietary fibers (Zhao et al. 2021). SCFAs not only provide energy to the intestinal epithelial cells but also regulate immune responses, inhibit inflammation, and promote the integrity of the gut barrier (Haskey et al. 2023). In addition, the gut microbiota can synthesize vitamins, promote the body's energy balance, and maintain metabolic homeostasis. Disruption of the gut microbiota often leads to the development of diseases such as obesity and hypertension (Ma and Lee 2025).

Diet plays a key role in regulating gut microbiota homeostasis. A plant‐based diet rich in polyphenols and fermentable dietary fibers can continuously promote the proliferation of bifidobacteria while inhibiting the growth of proteolytic bacteria, thus enhancing SCFA production and intestinal mucosal barrier function (Makki et al. 2018; Régnier et al. 2023). In contrast, ultra‐processed diets high in fats and sugars lead to characteristic dysbiosis, marked by an increase in Enterobacteriaceae and a decrease in Lactobacillaceae, raising the risk of diabetes and brain disorders (González Olmo et al. 2021; Aguayo‐Patrón and de la Calderón Barca 2017). To quantify these interactions, Kase and his research team developed the Dietary Index of Gut Microbiota (DI‐GM) through a systematic integration of existing literature. This index aims to quantitatively assess the impact of dietary patterns on gut microbiota (Kase et al. 2024). The index incorporates 14 dietary components with significant modulatory effects, categorizing them into beneficial components that promote microbiota health (e.g., whole grains, dietary fibers, fermented dairy products, broccoli, coffee, and polyphenol‐rich green tea) and restrictive components that may have negative effects (e.g., red meat, processed meats, refined carbohydrates, and high‐fat diets). The research team validated the scientific basis of the DI‐GM using dietary data from NHANES, confirming its ability to effectively reflect the relationship between dietary quality and biomarkers such as microbiota diversity and SCFA metabolism. While the DI‐GM provides a standardized framework based on evidence‐based medicine, no studies have yet explored its relationship with MetS.

This study, based on large‐scale representative samples from six cycles of NHANES conducted between 2007 and 2018, explores the relationship between DI‐GM and the risk of MetS. Furthermore, we also analyzed the mediating roles of albumin levels and the systemic inflammation index (SII) in this relationship, revealing the connection between DI‐GM and MetS from a nutrition–inflammation interaction perspective. This provides important reference value for formulating public health policies for the prevention and treatment of MetS.

## 2 Materials and Methods

### 2.1 Study Design and Participants

**FIGURE 1:**
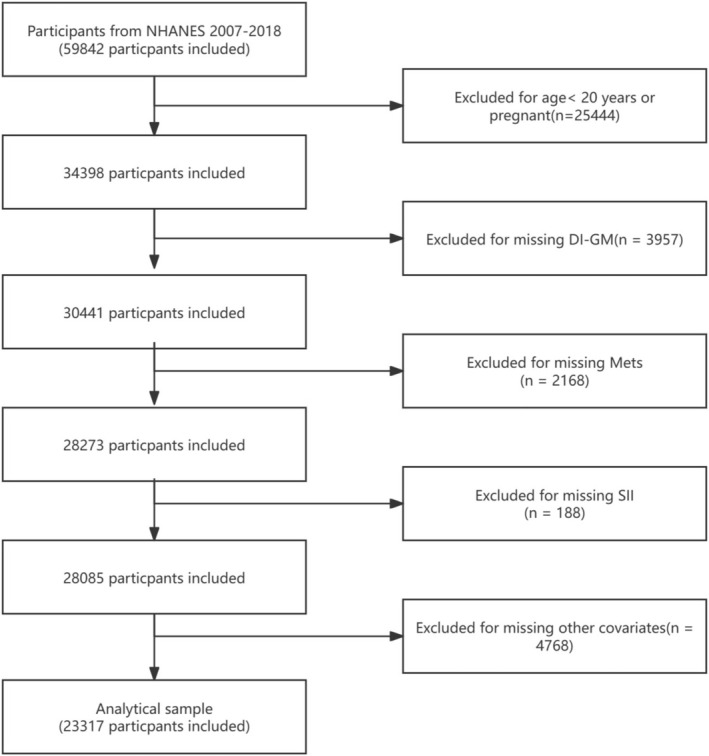
Flowchart of the study.

NHANES employs a stratified multistage sampling design, covering a broad population with representative samples from different age groups, genders, races, and regions. The data included health assessments, laboratory tests, nutritional status, as well as socioeconomic, lifestyle, and other information, widely used in public health research. All data have been anonymized to ensure participant privacy. The data used in this study were from the NHANES 2007–2018 dataset, including a total of 59,842 participants. During the data selection process, the following exclusion criteria were applied: (1) individuals aged < 20 years or pregnant (*n* = 25,444); (2) missing DI‐GM data (*n* = 3957); (3) missing MetS diagnostic data (*n* = 2168); (4) missing SII data (*n* = 188); (5) participants lacking other covariates (*n* = 4768). A detailed selection flowchart is shown in Figure 1.

### 2.2 Exposure and Outcome Variable Assessment

Kase et al. (2024) developed the DI‐GM index to quantify the impact of diet on gut microbiota health. This assessment system integrates 14 dietary indicators, including 10 positive regulatory components (avocados, broccoli, chickpeas, coffee, cranberries, fermented dairy products, fiber, green tea, soy products, whole grains) and 4 negative regulatory components (red meat, processed meats, refined grains, high‐fat diets). Data collection used the standardized 24‐h dietary recall method from NHANES, implemented through the automated multiple‐pass method (AMPM) developed by the USDA. All interviewers underwent uniform training to reduce recall bias. Each participant completed two independent dietary surveys, and the average value was used for calculation. The scoring mechanism was based on gender‐specific median thresholds: 1 point was given for a positive indicator intake above the median value, and 1 point was given for a negative indicator intake below the median value, with high‐fat diets having a threshold of 40% of total energy intake. The total score range is 0–14, with higher scores indicating a more favorable diet for gut microbiota health. Based on previous research, the scores were divided into four groups: 0–3, 4, 5, ≥ 6. The specific components, measurement standards, and scoring details are provided in Table S1. The clinical diagnosis of MetS is based on the metabolic abnormality evaluation criteria published by the National Cholesterol Education Program Adult Treatment Panel III (NCEP‐ATPIII). This criterion requires individuals to meet at least three out of five core metabolic parameters (abdominal obesity, elevated blood pressure, abnormal fasting glucose, reduced HDL cholesterol levels, and elevated triglyceride levels) to confirm a MetS diagnosis. Specific diagnostic criteria are detailed in Table S2.

### 2.3 Covariates and Mediators

We reviewed the literature and included relevant covariates, such as age, gender, race, educational background (less than high school, high school, more than high school), marital status (married or cohabiting, divorced/separated/widowed, and never married), poverty‐to‐income ratio (PIR) (< 2, ≥ 2), BMI (< 25.0 kg/m^2^, 25.0–29.9 kg/m^2^, > 29.9 kg/m^2^), smoking status (never, former, and current), and alcohol consumption (never, former, and current). Former smokers were those who had quit smoking and accumulated more than 100 cigarettes; current smokers were those who still smoke and had a total consumption of 100 or more cigarettes; non‐smokers were those who had never smoked or had smoked fewer than 100 cigarettes in total. Non‐drinkers were those who had consumed fewer than 12 alcoholic drinks in their lifetime, former drinkers were those who had consumed 12 or more alcoholic drinks in their lifetime but had not drunk alcohol in the past year, and current drinkers were those who had consumed 12 or more alcoholic drinks in their lifetime and had at least one alcoholic drink in the past year. Laboratory tests include aspartate aminotransferase (AST), alanine aminotransferase (ALT), total bilirubin (TBil), uric acid (UA), blood urea nitrogen (BUN), total cholesterol (TC), lactate dehydrogenase (LDH), and estimated glomerular filtration rate (eGFR), with eGFR calculated using the CKD‐EPI formula. To assess the potential issue of multicollinearity among the independent variables in the logistic regression models, we calculated the variance inflation factor (VIF) for all covariates included in the model (Figure S1). Albumin is one of the primary proteins in plasma and effectively reflects the body's nutritional status and liver function. Low albumin levels are often associated with insulin resistance, oxidative stress, and chronic low‐grade inflammation, making albumin an important indicator for studying the relationship between dietary factors and metabolic syndrome (Succurro et al. 2021). Chronic low‐grade inflammation is considered one of the key mechanisms underlying metabolic syndrome, and SII accurately reflects the body's inflammatory burden, thereby influencing key metabolic processes such as fat metabolism and insulin sensitivity (McPhee and Schertzer 2015). Therefore, this study selected albumin and SII as mediating indicators to further explore the association between DI‐GM and MetS. The calculation formulas for CKD‐EPI and SII are provided in Supporting Information.

### 2.4 Statistical Analysis

To ensure the representativeness of the samples, we followed the guidelines on weights provided in the NHANES documentation. Specifically, we applied sampling weights and accounted for primary sampling units and stratification factors in the data analysis. We used a laboratory weight of 1/6 in all laboratory test data analyses to ensure accurate estimates reflecting the U.S. population. During the data analysis, *t*‐tests were used for normally distributed variables, and Wilcoxon rank‐sum tests were applied for non‐normally distributed variables. For categorical variables, chi‐square tests were used. Logistic regression analysis was used to calculate the weighted odds ratios (OR) and 95% confidence intervals (95% CI) for the association between DI‐GM and MetS. The P for trend was obtained through a linear trend test by treating the DI‐GM categorical variables as continuous variables within the regression model. Model 1 did not adjust for any covariates, Model 2 adjusted for age, gender, and race, and Model 3 further adjusted for educational background, marital status, PIR, BMI, smoking status, alcohol consumption, eGFR, AST, ALT, TBil, UA, BUN, TC, and LDH. We explored the nonlinear relationship between DI‐GM and metabolic syndrome (MetS) using restricted cubic spline (RCS) regression analysis via the “rms” package, supplemented by piecewise logistic regression analysis to identify potential turning points. Four knots were placed at the 5th, 35th, 65th, and 95th percentiles of the DI‐GM distribution, in accordance with standard RCS modeling guidelines. The reference value for the spline model was set at the median of the DI‐GM distribution. Additionally, we analyzed different subgroups (age, gender, race, BMI, smoking status, alcohol consumption) to examine the stability of the results. Finally, we conducted mediation analysis using the “mediation” package in R with stepwise regression and 1000 bootstrap resampling iterations to assess the mediating role of albumin and SII levels in the relationship between DI‐GM and MetS. The causal pathway assumptions (DI‐GM → albumin/SII → MetS) were based on established biological mechanisms linking dietary quality, systemic inflammation, and metabolic health. However, given the cross‐sectional design of the study, the causal interpretation of these pathways should be made with caution. To validate the robustness and consistency of the study results, we conducted several comprehensive sensitivity analyses. First, to verify the reliability of the study results, we performed repeated analyses using an unweighted approach. Second, we performed multiple imputation for missing covariates in the NHANES data using the chained equation method. To address missing baseline data, we conducted multiple imputation based on five imputed datasets and repeated analyses on the imputed data to ensure results were not confounded by missing information. Third, BMI is highly correlated with the core components of the MetS diagnostic criteria. To assess whether adjusting for BMI could lead to over‐adjustment and affect the association between DI‐GM and MetS, we repeated the analysis after excluding BMI as a covariate. All analyses were performed using Empower 4.2 and R 4.2.0, with *p* < 0.05 set as the significance threshold.

## 3 Results

### 3.1 Participant Characteristics

A total of 23,317 participants were included in this study, with an average age of 47.29 years, and 50.03% of the participants were male. Among all participants, 6414 were diagnosed with MetS, accounting for 27.51%. As shown in Table 1, compared to the non‐MetS group, participants in the MetS group had the following characteristics: older age, higher proportion of females, higher proportion of non‐Hispanic White participants, lower education levels, more participants with a marital status of divorced/separated/widowed, lower PIR, higher BMI, and higher proportions of individuals with a history of alcohol consumption and smoking.

**TABLE 1:** Characteristics at baseline of participants grouped by MetS status.

MetS	Total (*n* = 23,317)	Without MetS (*n* = 16,903)	With MetS (*n* = 6414)	*p*
Age, years	47.29 (46.78, 47.80)	44.63 (44.09, 45.17)	55.27 (54.69, 55.85)	< 0.001
*Sex*				< 0.001
Male	11,666 (50.03%)	8654 (51.20%)	3012 (46.96%)	
Female	11,651 (49.97%)	8249 (48.80%)	3402 (53.04%)	
*Race*				< 0.001
Mexican American	3462 (14.85%)	2410 (14.26%)	1052 (16.40%)	
Other Hispanic	2331 (10.00%)	1697 (10.04%)	634 (9.88%)	
Non‐Hispanic White	10,362 (44.44%)	7357 (43.52%)	3005 (46.85%)	
Non‐Hispanic Black	4650 (19.94%)	3386 (20.03%)	1264 (19.71%)	
Other race	2512 (10.77%)	2053 (12.15%)	459 (7.16%)	
*Education level*				< 0.001
Under high school	5228 (22.42%)	3448 (20.40%)	1780 (27.75%)	
High school or equivalent	5340 (22.90%)	3745 (22.16%)	1595 (24.87%)	
Above high school	12,749 (54.68%)	9710 (57.45%)	3039 (47.38%)	
*Marital status*				< 0.001
Married/Living with partner	13,988 (59.99%)	10,071 (59.58%)	3917 (61.07%)	
Divorced/Separated/Widowed	5085 (21.81%)	3258 (19.27%)	1827 (28.48%)	
Never married	4244 (18.20%)	3574 (21.14%)	670 (10.45%)	
*PIR*				< 0.001
< 2	11,030 (47.30%)	7741 (45.80%)	3289 (51.28%)	
≥ 2	12,287 (52.70%)	9162 (54.20%)	3125 (48.72%)	
*BMI*				< 0.001
< 25.0 kg/m^2^	6569 (28.17%)	6176 (36.54%)	393 (6.13%)	
25.0–29.9 kg/m^2^	7644 (32.78%)	5867 (34.71%)	1777 (27.71%)	
> 29.9 kg/m^2^	9104 (39.04%)	4860 (28.75%)	4244 (66.17%)	
*Drinking status*				< 0.001
Never	3139 (13.46%)	2128 (12.59%)	1011 (15.76%)	
Former	3639 (15.61%)	2188 (12.94%)	1451 (22.62%)	
Now	16,539 (70.93%)	12,587 (74.47%)	3952 (61.62%)	
*Smoking status*				< 0.001
Never	12,836 (55.05%)	9580 (56.68%)	3256 (50.76%)	
Former	5700 (24.45%)	3728 (22.06%)	1972 (30.75%)	
Now	4781 (20.50%)	3595 (21.27%)	1186 (18.49%)	
eGFR, mL/min/1.73 m^2^	94.34 (93.68, 95.00)	96.90 (96.18, 97.62)	86.65 (85.89, 87.41)	< 0.001
ALT, U/L	25.42 (25.12, 25.73)	24.44 (24.12, 24.75)	28.39 (27.63, 29.14)	< 0.001
AST, U/L	25.33 (25.10, 25.57)	24.98 (24.73, 25.22)	26.40 (25.84, 26.96)	< 0.001
TBil, mg/dL	0.67 (0.66, 0.68)	0.67 (0.66, 0.69)	0.64 (0.63, 0.65)	< 0.001
UA, mg/dL	5.45 (5.42, 5.48)	5.28 (5.25, 5.32)	5.96 (5.91, 6.00)	< 0.001
BUN, mg/dL	13.66 (13.51, 13.80)	13.20 (13.05, 13.35)	15.03 (14.78, 15.27)	< 0.001
TC, mg/dL	194.09 (193.07, 195.11)	194.44 (193.42, 195.47)	193.02 (191.30, 194.74)	0.085
LDH, mg/dL	132.23 (131.46, 133.00)	130.80 (130.02, 131.58)	136.52 (135.25, 137.79)	< 0.001

*Note:* Data are expressed as median (interquartile range) or counts (weighted proportions).

Abbreviations: ALT, alanine aminotransferase; AST, aspartate aminotransferase; BMI, body mass index; BUN, blood urea nitrogen; eGFR, estimated glomerular filtration rate; LDH, Lactate Dehydrogenase; PIR, family income‐to‐poverty ratio; TBil, Total Bilirubin; TC, total cholesterol; UA, uric acid.

### 3.2 Association Between DI‐GM and MetS

**FIGURE 2:**
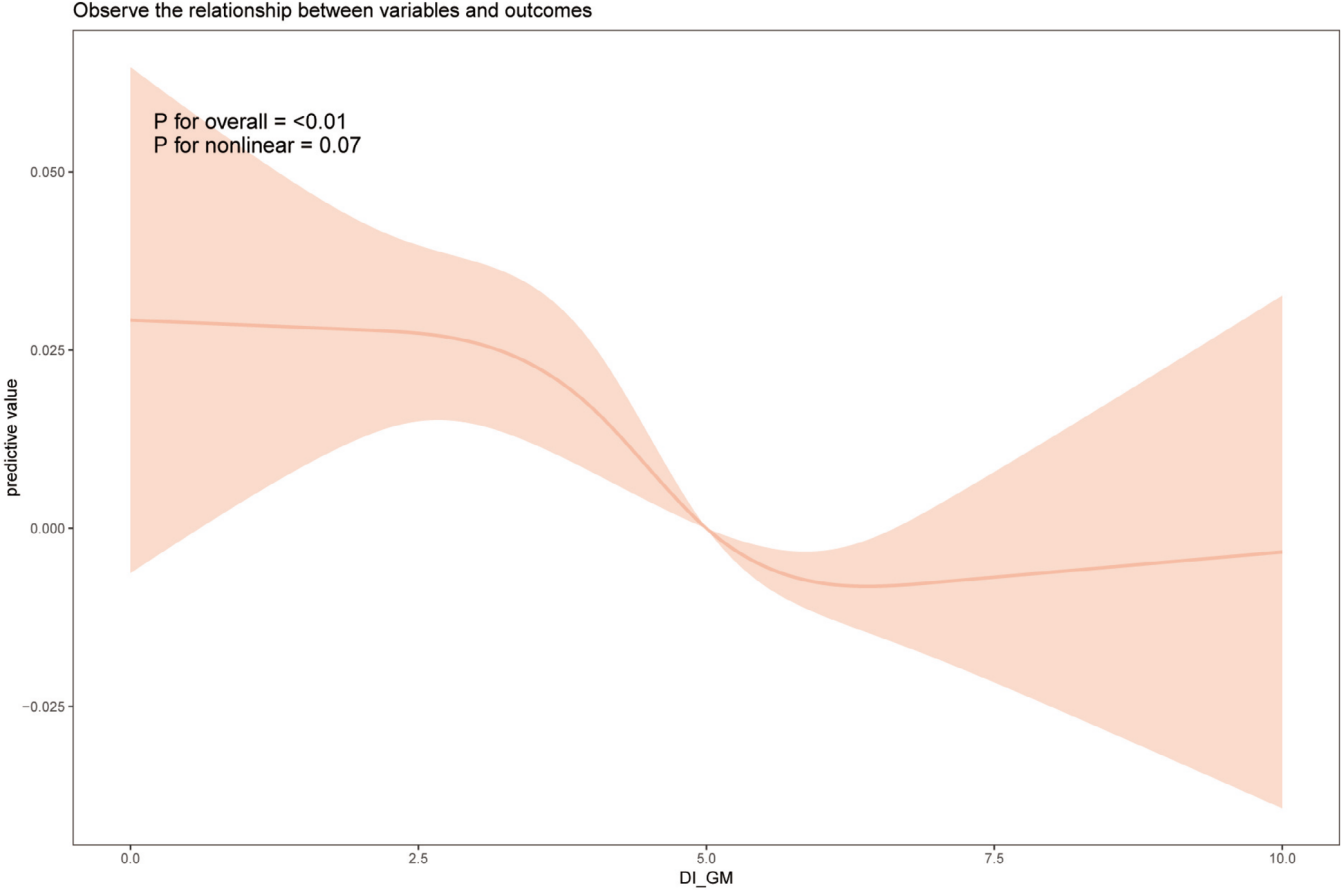
Weighted restricted cubic spline analysis of the association between gut microbiota (DI‐GM) and metabolic syndrome; the shaded area represents the 95% confidence interval, adjusted for age, gender, race, education level, marital status, PIR, BMI, drinking status, smoking status, eGFR, AST, ALT, TBil, UA, BUN, TC, and LDH.

Table 2 presents the results of the multivariable weighted logistic regression, showing a strong negative correlation between DI‐GM and the incidence of MetS. In the fully adjusted model, for each 1‐point increase in DI‐GM, the risk of MetS decreased by 0.053 (OR = 0.947 [0.921, 0.974]). As DI‐GM increased, this negative association became more significant. Compared to the group with the lowest DI‐GM score (0–3), the group with a score of 5 had a 0.192 lower risk of MetS (OR = 0.808 [0.707, 0.923]), and the group with a score of ≥ 6 had a 0.204 lower risk (OR = 0.796 [0.700, 0.905]). The weighted RCS analysis revealed an approximate nonlinear association between DI‐GM and MetS (nonlinear *p* = 0.07) (Figure 2). Piecewise logistic regression showed that when DI‐GM was above 3, there was a significant negative correlation between DI‐GM and the risk of MetS (OR = 0.937 [0.924, 0.972]). However, when DI‐GM was below 3, the association between DI‐GM and MetS risk was not significant (OR = 0.996 [0.911, 1.089]), although the OR was still less than 1 (Table S3).

**TABLE 2:** The association between DI‐GM and MetS.

Characteristics	Model 1 OR (95% CI)	Model 2 OR (95% CI)	Model 3 OR (95% CI)
Continuous DI_GM (*n* = 23,317)	0.933 (0.909, 0.957) < 0.001	0.890 (0.867, 0.913) < 0.001	0.947 (0.921, 0.974) < 0.001
*DI‐GM group*			
0–3 (*n* = 5614)	1	1	1
4 (*n* = 5693)	0.890 (0.792, 0.999) 0.051	0.875 (0.773, 0.990) 0.037	0.950 (0.816, 1.106) 0.511
5 (*n* = 5387)	0.796 (0.714, 0.889) < 0.001	0.716 (0.638, 0.804) < 0.001	0.808 (0.707, 0.923) 0.003
≥ 6 (*n* = 6623)	0.751 (0.669, 0.843) < 0.001	0.619 (0.552, 0.695) < 0.001	0.796 (0.700, 0.905) < 0.001
*p* for trend	< 0.001	< 0.001	< 0.001

*Note:* Model 1: Unadjusted for any covariates. Model 2: Adjusted for age, gender, and race. Model 3: Adjusted for age, gender, race, education level, marital status, PIR, BMI, Drinking status, Smoking status, eGFR, AST, ALT, TBil, UA, BUN, TC and LDH.

Abbreviations: CI, confidence interval; DI‐GM, dietary index for gut microbiota; OR, odds ratio.

Table S4 showed a significant inverse relationship between DI‐GM and several components of MetS, particularly for elevated BP, abnormal FBG, and lower HDL. Specifically, a higher DI‐GM score was associated with a lower risk of abdominal obesity, elevated BP, abnormal FBG, and lower HDL, with P for trend values all below 0.05, suggesting a consistent trend. The most pronounced associations were observed in the groups with DI‐GM scores ≥ 5, where the risk of abdominal obesity and elevated BP was significantly reduced.

### 3.3 Subgroup Analysis

**FIGURE 3:**
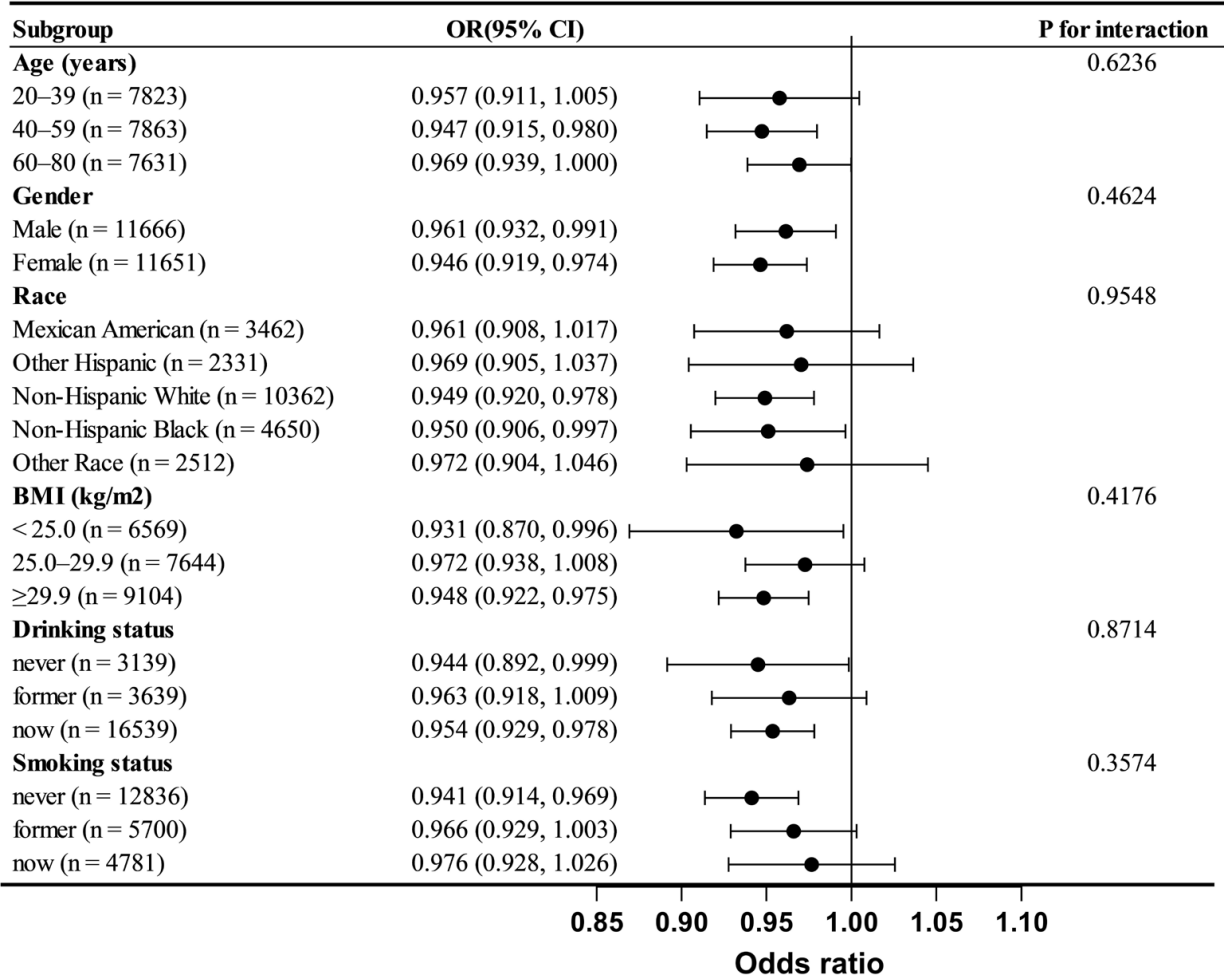
Subgroup analysis of the association between gut microbiota (DI‐GM) and metabolic syndrome, adjusted for age, gender, race, education level, marital status, PIR, BMI, drinking status, smoking status, eGFR, AST, ALT, TBil, UA, BUN, TC, and LDH.

We conducted a subgroup analysis based on factors such as age, gender, race, BMI, alcohol consumption, and smoking status to investigate whether the association between DI‐GM and MetS remained consistent across different subgroups. The results indicated that although the confidence intervals crossed 1 for the 20–39 age group, Mexican American, Other Hispanic, Other Race, BMI between 25.0 and 29.9, former drinkers, former smokers, and current smokers, the OR values were still below 1. Furthermore, a significant negative correlation between DI‐GM and MetS was observed in most subgroups. Notably, no significant interactions were found in any subgroup (interaction *p* > 0.05), suggesting that the association between DI‐GM and MetS is stable across these factors (Figure 3).

### 3.4 Association Between Albumin, SII, DI‐GM, and MetS

The multivariable weighted logistic regression between DI‐GM and albumin, as well as SII, is shown in Table 3. In the fully adjusted model, DI‐GM was positively correlated with albumin (*β* = 0.091 [0.057, 0.124]) and negatively correlated with SII (*β* = −7.837 [−11.050, −4.625]). Table 4 presents the association between albumin, SII, and MetS after multivariable weighted logistic regression. In the fully adjusted model, albumin was negatively associated with the risk of MetS (OR = 0.957 [0.943, 0.972]), while SII was positively associated with the risk of MetS (OR = 1.001 [1.000, 1.003]).

**TABLE 3:** The association between DI‐GM and albumin and SII.

Characteristics	*β*	95% CI	*p*
*Albumin*			
Model 1	0.108	0.067, 0.14	< 0.001
Model 2	0.160	0.124, 0.197	< 0.001
Model 3	0.091	0.057, 0.124	< 0.001
*SII*			
Model 1	−6.880	−9.927, −3.834	< 0.001
Model 2	−10.659	−13.848, −7.470	< 0.001
Model 3	−7.837	−11.050, −4.625	< 0.001

*Note:* Model 1: Unadjusted for any covariates. Model 2: Adjusted for age, gender, and race. Model 3: Adjusted for age, gender, race, education level, marital status, PIR, BMI, Drinking status, Smoking status, eGFR, AST, ALT, TBil, UA, BUN, TC and LDH.

Abbreviation: CI, confidence interval.

**TABLE 4:** The association between MetS and albumin and SII.

Characteristics	OR	95% CI	*p*
*Albumin*			
Model 1	0.947	0.921, 0.974	< 0.001
Model 2	0.903	0.891, 0.916	< 0.001
Model 3	0.957	0.943, 0.972	< 0.001
*SII*			
Model 1	1.000	1.000, 1.000	< 0.001
Model 2	1.002	1.001, 1.004	< 0.001
Model 3	1.001	1.000, 1.003	< 0.001

*Note:* Adjusted for age, gender, race, education level, marital status, PIR, BMI, Drinking status, Smoking status, eGFR, AST, ALT, TBil, UA, BUN, TC and LDH.

Abbreviations: 95% CI, 95% confidence interval; OR, odds ratio.

### 3.5 Mediating Role of Albumin and SII

**FIGURE 4:**
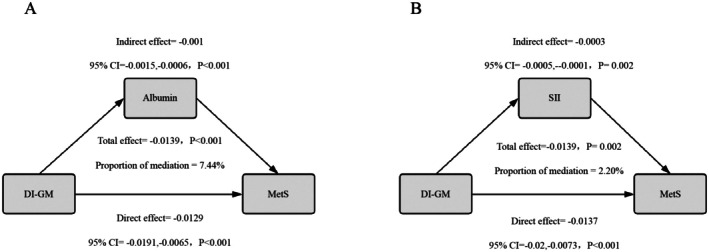
Mediation analysis of the effects of albumin (A) and systemic immune‐inflammation index (SII) (B) on the relationship between the gut microbiota dietary index (DI‐GM) and metabolic syndrome.

We further conducted mediation analysis to explore the potential mechanisms through which DI‐GM affects the incidence of MetS. After adjusting for all covariates, a significant association between DI‐GM and MetS was found, with a total effect coefficient of −0.0139 (*p* < 0.001). The indirect effect of albumin was −0.001 (*p* < 0.001), and the mediation effect accounted for 7.44% of the total effect (*p* < 0.001). The indirect effect of SII was −0.0003 (*p* = 0.002), and the mediation effect accounted for 2.20% of the total effect (*p* = 0.002; Figure 4; Table S5).

### 3.6 Dietary Pattern Differences Across MetS Statuses

To enhance the practical interpretability of the research findings, this study combined food intake data from the Food Frequency Questionnaire to further explore differences in DI‐GM dietary components between individuals with metabolic syndrome (MetS) and those without MetS, and analyzed the associations between specific food components and MetS. The results showed that compared with the non‐MetS group, the MetS group had lower daily intake of foods beneficial to gut health, particularly in avocados, cauliflower, chickpeas, cranberries, fermented dairy products, fiber, soy, and whole grains. Additionally, the MetS group had a higher intake of processed meats, coffee, and green tea, while the intake of fat and refined grains was lower (Figure S2). Further weighted logistic regression results showed that chickpeas (OR = 0.997 [0.994, 1.000]), coffee (OR = 1.000 [0.999, 1.000]) were significantly negatively associated with MetS risk, while refined grains (OR = 1.023 [1.011, 1.036]), processed meat (OR = 1.056 [1.031, 1.082]), and red meat (OR = 1.027 [1.011, 1.044]) were significantly positively associated with MetS risk (Table S6).

### 3.7 Sensitivity Analysis

In the unadjusted model, the negative correlation between DI‐GM and MetS risk remained significant (Table S7). In the fully adjusted model, each 1‐standard deviation increase in DI‐GM was associated with a 0.046% reduction in MetS risk (OR = 0.954 [0.934, 0.974]). Quartile analysis results indicate that this association still exhibits a dose–response relationship: compared with the lowest DI‐GM score group (0–3 points), the group with a score of 5 points had a 0.118 reduction in MetS risk (OR = 0.882 [0.801, 0.972]), while the group with DI‐GM scores ≥ 6 had a risk reduction of 0.16 (OR = 0.84 [0.765, 0.923]). This association remained stable after multiple imputation. In the fully adjusted model, each 1‐standard deviation increase in DI‐GM was associated with a 0.046 reduction in MetS risk (OR = 0.954 [0.931, 0.976]). Quartile analysis results showed that compared with the lowest DI‐GM score group (0–3 points), the group with a score of 5 had a 0.127 reduction in metabolic syndrome risk (OR = 0.873 [0.773, 0.986]), while the group with a DI‐GM score ≥ 6 had a risk reduction of 0.188 (OR = 0.812 [0.724, 0.912]; Table S8). After removing BMI from the model's adjusted covariates, each 1 standard deviation increase in DI‐GM was associated with a 0.085 reduction in the risk of metabolic syndrome (OR = 0.915 [0.892, 0.939]). Quartile analysis showed that, compared to the lowest DI‐GM score group (0–3 points), the group with a score of 5 had a 0.242 lower risk of metabolic syndrome (OR = 0.758 [0.670, 0.857]), while the group with a DI‐GM score ≥ 6 had a 0.311 lower risk (OR = 0.689 [0.612, 0.776]) (Table S9).

## 4 Discussion

Our findings indicate that the DI‐GM index is significantly negatively correlated with MetS. Subgroup analysis and interaction tests did not reveal any confounding factors that could affect this association. RCS analysis further supports this negative correlation, suggesting that when DI‐GM is increased to at least 3, the risk of MetS can be significantly reduced. We further observed the mediating role of albumin and SII in this association, which provides important theoretical support for future interventions. Our study offers a new perspective for the formulation of public health policies, emphasizing the importance of dietary structure and its impact on metabolic health.

Gut microbiota plays a key role in the body's metabolic processes. Changes in the composition and function of the microbiota can lead to the development of metabolic syndrome by affecting the host's metabolism and immune regulation. The abnormal ratio of Firmicutes to Bacteroidetes is a key biomarker for obesity and related metabolic syndromes. In obese individuals, the gut microbiota shows a typical “Firmicutes‐dominant” profile, with the Firmicutes/Bacteroidetes ratio significantly higher than that in healthy individuals. This microbial imbalance may lead to excessive weight gain and metabolic disorders by enhancing the host's energy intake efficiency and promoting fat storage (Louis et al. 2016; Cui et al. 2022). *Akkermansia muciniphila* , an important regulator of gut barrier function, has been shown to be closely associated with the development of metabolic diseases, such as insulin resistance, type 2 diabetes, and obesity, when its abundance decreases (Zhang et al. 2021; García‐Sanmartín et al. 2022). Probiotic interventions that restore the colonization levels of this strain can significantly improve the host's glucose and lipid metabolism indicators (Depommier et al. 2019). The enhanced colonization of Bifidobacterium and Lactobacillus has also been shown to have multiple metabolic protective effects. These strains not only effectively improve gut barrier integrity but also inhibit chronic inflammatory responses by regulating pathways such as TLR4/NF‐κB, thus playing an important role in the prevention and treatment of metabolic syndrome (Kang et al. 2022; Liu, Zhao, et al. 2022; Yue et al. 2023).

The metabolic products of gut microbiota play an important role in regulating the host's metabolism and immune function. SCFAs are key metabolites produced by gut microbiota through the fermentation of dietary fibers. SCFA levels are typically lower in individuals with obesity and diabetes compared to healthy individuals, and the lack of SCFAs may be a significant factor in metabolic disorders (Jayakumar and Loomba 2019). Studies have shown that SCFAs bind to G‐protein coupled receptors (such as GPR41 and GPR43), activating various signaling pathways that affect lipid metabolism, glucose metabolism, and inflammation (Pant et al. 2023). Additionally, SCFAs regulate body weight by inhibiting appetite and promoting energy expenditure, further impacting metabolic health (Baryła et al. 2022). Trimethylamine‐N‐oxide (TAMO), a gut microbiota metabolite of red meat, has been shown to be elevated in individuals with cardiovascular diseases, diabetes, obesity, and other metabolic disorders (Syu et al. 2023). Barrea et al. (Barrea et al. 2018) found a positive correlation between TAMO levels and the risk of MetS, suggesting that TAMO may serve as an early predictor for MetS. Moreover, gut microbiota play a key role in synthesizing essential vitamins such as vitamin B12, vitamin K, and folate, which are crucial for maintaining the host's metabolic homeostasis and immune balance (Ser et al. 2023). Among these, vitamin B12 acts as a core cofactor for methionine synthase and methylmalonyl‐CoA mutase, playing a dual role in maintaining the one‐carbon metabolic cycle and the tricarboxylic acid cycle. It influences the host's metabolic state by regulating methylation reactions and cellular energy metabolism (McCorvie et al. 2023; Lyon et al. 2020; Griffith et al. 2025).

Diet is a key factor influencing the composition and function of the gut microbiota. Dietary components significantly affect the microbial community structure, leading to differential changes in the abundance of specific microbiota. For example, fiber‐rich plant‐based diets can increase the abundance of Bacteroidetes and SCFA‐producing bacteria (such as members of the genus Faecalibacterium), whereas high‐fat/high‐sugar diets are often associated with decreased gut microbiota diversity, further contributing to gut dysbiosis and chronic low‐grade inflammation (Jacky et al. 2023; Bundgaard‐Nielsen et al. 2020; Minaya et al. 2021). Diet can influence the host's immune regulation, gut barrier function, and overall metabolic network by altering the microbiota. Potential mechanisms include SCFA signaling, bile acid metabolism, and other pathways (Liu, Yang, et al. 2022; Xu et al. 2023). The DI‐GM index developed by Kase and his team can quantify the effect of diet on gut microbiota to some extent. A higher DI‐GM index suggests a more favorable dietary impact on gut microbiota homeostasis (Kase et al. 2024).

Our study found a significant negative correlation between the DI‐GM index and MetS risk. This association became more pronounced when the DI‐GM index exceeded 3, and the correlation strengthened as DI‐GM increased. Subgroup analysis showed no significant interaction effects between DI‐GM and MetS risk across different subgroups, underscoring the stability of this association. Additionally, we identified the mediating role of albumin and SII in the relationship between DI‐GM and MetS risk. This mediation effect provides important insights into the biological mechanisms through which dietary diversity influences MetS. Albumin, as a key plasma protein, plays multiple physiological roles, including involvement in nutritional status, inflammatory responses, and antioxidant functions (Soeters et al. 2019; Awasthi et al. 2019). In addition to maintaining plasma colloid osmotic pressure, albumin contributes to improving endothelial function and enhancing insulin sensitivity (Liu et al. 2023; Alzayadneh et al. 2023). Adequate protein intake is closely associated with albumin synthesis, and the dietary diversity emphasized by the DI‐GM index may optimize amino acid intake, further promoting hepatic albumin production. On the other hand, SII serves as a marker reflecting systemic immune and inflammatory status, with elevated levels closely linked to metabolic disorders and chronic diseases. A highly diverse diet provides abundant polyphenols, dietary fibers, and other anti‐inflammatory components, which may exert their effects by inhibiting inflammatory pathways such as NF‐κB (Ma et al. 2022). Furthermore, dietary diversity may regulate gut microbiota composition and influence the release of inflammatory cytokines, such as TNF‐α and IL‐6, thereby exacerbating metabolic dysregulation (Shivappa et al. 2014). Dysbiosis of the gut microbiota may trigger the release of endogenous lipopolysaccharides (LPS), leading to systemic inflammatory responses, which are considered a key pathological mechanism underlying MetS (Chang et al. 2019). Notably, albumin and SII may interact synergistically to influence metabolic homeostasis. Chronic inflammatory states may accelerate albumin catabolism, while high albumin levels can help mitigate inflammatory responses (Tabata et al. 2021; Cai et al. 2022). This bidirectional interaction suggests that dietary interventions may not only improve nutritional status but also regulate inflammatory responses, potentially exerting a synergistic metabolic protective effect.

Analyses of individual dietary components provide additional insights. Compared with participants without MetS, those with MetS consumed fewer avocados, cruciferous vegetables, legumes, cranberries, fermented dairy products and whole grains, and more processed meats and refined grains. In weighted logistic regression models, chickpeas were inversely associated with MetS risk, whereas refined grains, processed meat and red meat were positively associated. These findings echo earlier reports that diets high in fiber and plant polyphenols foster a diverse microbiota that produces SCFAs and other beneficial metabolites, whereas diets rich in saturated fat and processed meat promote dysbiosis, inflammation and metabolic dysfunction (Nemzer et al. 2025; Suriano et al. 2022). They also align with observational studies linking ultra‐processed food intake to obesity and metabolic syndrome (Canhada et al. 2023). Coffee intake showed a marginal inverse association with MetS risk in our data, in agreement with recent studies, suggesting that bioactive compounds such as chlorogenic acids may modulate the microbiota–host axis (Rosa et al. 2024).

The strengths of this study lie in its large sample size, long time span, and its novel investigation into the association between the DI‐GM index and MetS, providing a new perspective for the development of future public health policies. It emphasizes the importance of dietary structure and its impact on metabolic health. However, the study has several limitations. The research sample was derived from the United States, where there are significant regional differences in dietary culture, genetic backgrounds, and environmental exposures. Therefore, the generalizability of the findings to other regions may be influenced. Although we included as many covariates as possible to reduce confounding bias, metabolic syndrome is a complex disease involving multiple factors and interactions, and we cannot completely rule out the influence of some potential confounders, and the characteristics of the excluded population may differ from those of the included participants, which could introduce selection bias. Additionally, the dietary assessment was based on self‐reports, which may be subject to recall bias and subjective reporting; although this method is widely used in NHANES, it may not accurately capture long‐term dietary habits. No internal consistency analysis was performed, and the DI‐GM score was not validated against other dietary assessment tools, such as the Food Frequency Questionnaire, and we acknowledge that the potential interactions or synergistic effects between individual dietary components may have been overlooked in the analysis, which could influence the overall association between DI‐GM and MetS. Due to the inherent limitations of cross‐sectional studies, this study cannot infer a causal relationship between DI‐GM and MetS. Although the findings suggest that optimizing dietary structure may be beneficial for the prevention and treatment of MetS, these conclusions require further validation through prospective cohort studies or intervention trials. Such validation will enhance the clinical translational value of the findings.

## 5 Conclusions

Summary, this study reveals a significant negative correlation between the DI‐GM index and MetS, with albumin and SII playing a mediating role in this association. These findings emphasize the importance of dietary structure adjustments and provide a new perspective for improving clinical management and public health interventions for MetS. Considering the cultural, genetic, and metabolic diversity among populations, the findings of this study should be interpreted with caution when generalizing to other regions. Moreover, due to the inherent limitations of the cross‐sectional study design, these results should be viewed with caution. Further validation through prospective cohort studies and intervention trials is warranted.

## Author Contributions

**Shouxin Wei:** conceptualization, methodology, formal analysis, software, writing – Original Draft. **Sijia Yu:** conceptualization, software, formal analysis, writing – review and editing. **Chuan Qian:** data curation, methodology, writing – review and editing. **Shouxin Wei** and **Sijia Yu** are the co‐first authors of this study.

## Conflicts of Interest

The authors declare no conflicts of interest.

## Supporting information


**Figure S1:** Variance inflation factor values of covariates in this study.
**Figure S2:** Average intake of DI‐GM dietary components.
**Table S1:** Components and scoring criteria of DI‐GM.
**Table S2:** Diagnostic criteria for metabolic syndrome.
**Table S3:** Threshold effect analysis of DI‐GM and MetS associations based on a two‐segment linear regression model.
**Table S4:** The association between DI‐GM and the individual components of MetS.
**Table S5:** Analysis of the mediating role of albumin and SII in the association between DI‐GM and metabolic syndrome.
**Table S6:** The association between dietary components in DI‐GM and MetS.
**Table S7:** The association between DI‐GM and MetS in the unweighted model.
**Table S8:** The association between DI‐GM and MetS after multiple interpolation.

## Data Availability

The dataset(s) supporting the conclusions of this article is available in the NHANES repository (https://www.cdc.gov/nchs/nhanes/index.htm, accessed 02/24/2025). Statistical analysis code will be made available upon request. Raw data supporting the conclusions of this paper are available from the corresponding authors upon request.
