# Supplementary material for: Association Between the Dietary Index for Gut Microbiota and Metabolic Syndrome: Mediation Effects of Albumin and Systemic Immune‐Inflammation Index
Source: Food Sci Nutr. 2025 Nov 11;13(11):e71194. doi: 10.1002/fsn3.71194 (PMC12603787; doi:10.1002/fsn3.71194)
Supplement: Supplementary file 1 — Figure S1: Variance inflation factor values of covariates in this study. Figure S2: Average intake of DI‐GM dietary components. Table S1: Components and scoring criteria of DI‐GM. Table S2: Diagnostic criteria for metabolic syndrome. Table S3: Threshold effect analysis of DI‐GM and MetS associations based on a two‐segment linear regression model. Table S4: The association between DI‐GM and the individual components of MetS. Table S5: Analysis of the mediating role of albumin and SII in the association between DI‐GM and metabolic syndrome. Table S6: The association between dietary components in DI‐GM and MetS. Table S7: The association between DI‐GM and MetS in the unweighted model. Table S8: The association between DI‐GM and MetS after multiple interpolation. [file FSN3-13-e71194-s001.zip › fsn371194-sup-0003-TableS1-S9-FigureS1-S2@Supplementary material.docx]

**Formula for calculating GFR,**

**Chronic Kidney Disease Epidemiology Collaboration(CKD-EPI):**

GFR = 141 × min(Scr/κ, 1)α × max(Scr/κ, 1)^ − 1.209 × 0.993^Age × 1.018 [if female] × 1.159 [if black people], where Scr is serum creatinine, κ is 0.7 for females and 0.9 for males, α is −0.329 for females and −0.411 for males. The min function indicates the minimum of Scr/κ or 1, and the max function indicates the maximum of Scr/κ or 1.

**Formula for calculating SII,**

SII= Platelet count × Neutrophil count/Lymphocyte count

**Supplementary Table 1.** Components and scoring criteria of DI-GM.

| Components of DI-GM | Food and nutrients. | Scoring standards |
| --- | --- | --- |
| Beneficial to gut microbiota | Avocados | For each component that benefits the gut microbiota, a score of 1 is assigned if the intake is equal to or greater than the sex-specific median; otherwise, the score is 0. |
|  | Broccoli |  |
|  | Chickpeas |  |
|  | Coffee |  |
|  | Cranberries |  |
|  | Fermented dairy (including yogurt, cheese, kefir, sour cream, buttermilk) |  |
|  | Fiber |  |
|  | Green tea |  |
|  | Soybean (including Soy milk, Tofu) |  |
|  | Whole grains (grains defined as whole grains, containing the entire grain kernel—the bran, germ, and endosperm) |  |
| Harmful to the gut microbiota. | High-fat diet (% energy) | A score of 0 is given if the intake is at or above 40% of total energy from fat; otherwise, the score is 1.For each other component that is detrimental to the gut microbiota, a score of 0 is assigned if the intake is equal to or greater than the sex-specific median; otherwise, the score is 1. |
|  | Processed meat (including frankfurters, sausages, corned beef, and luncheon meat that are made from beef, pork, or poultry) |  |
|  | Red meat (including beef, veal, pork, lamb, and game meat; excludes organ meat and cured meat) |  |
|  | Refined grains (refined grains that do not contain all of the components of the entire grain kernel) |  |

DI-GM,Dietary index for gut microbiota.

**Supplementary Table 2.** Diagnostic criteria for metabolic syndrome.

| Parameters | Criteria |
| --- | --- |
| Glucose | Fasting glucose ≥5.6 mmol/L (100 mg/dL) or drug treatment for elevated blood glucose |
| HDL cholesterol | <1.0 mmol/L (40 mg/dL) (men); <1.3 mmol/L (50 mg/dL) (women) or drug treatment for low HDL cholesterol |
| Triglycerides | ≥1.7 mmol/L (150 mg/dL) or drug treatment for elevated triglycerides |
| Obesity | Waist ≥102 cm (men) or ≥88 cm (women) |
| Hypertension | ≥130/85 mmHg or drug treatment for hypertension |

HDL,High-Density Lipoprotein.

**Supplementary Table 3.** Threshold effect analysis of DI-GM and MetS associations based on a two-segment linear regression model.

|  | Adjusted OR (95% CI) |
| --- | --- |
| Fitting by the standard linear model | 0.947 (0.921, 0.974) |
| Inflection point | 3 |
| DI-GM < 3 | 0.996 (0.911, 1.089) |
| DI-GM  >3 | 0.937 (0.924, 0.972) |
| *P* for Log-likelihood ratio | 0.129 |

**Supplementary Table 4.** The association between DI-GM and the individual components of MetS.

| Characteristics | Abdominal obesity OR (95% CI) | Elevated BP OR (95% CI) | Abnormal FBG OR (95% CI) | Lower HDL OR (95% CI) | Elevated TG OR (95% CI) |
| --- | --- | --- | --- | --- | --- |
| Continuous DI_GM (n = 23317) | 0.943 (0.901, 0.987) 0.014 | 0.946 (0.921, 0.972) <0.001 | 0.957 (0.931, 0.984) 0.003 | 0.961 (0.936, 0.986) 0.004 | 0.965 (0.933, 0.999) 0.0482 |
| DI-GM group |  |  |  |  |  |
| 0–3 (n=5614) | 1[Ref] | 1[Ref] | 1[Ref] | 1[Ref] | 1[Ref] |
| 4 (n=5693) | 0.906 (0.747, 1.098) 0.317 | 0.881 (0.772, 1.004) 0.062 | 1.001 (0.887, 1.129) 0.989 | 0.933 (0.832, 1.047) 0.244 | 1.105 (0.954, 1.281) 0.188 |
| 5 (n=5387) | 0.743 (0.602, 0.917) 0.007 | 0.830 (0.730, 0.944) 0.006 | 0.877 (0.777, 0.990) 0.038 | 0.827 (0.737, 0.929) 0.002 | 0.978 (0.832, 1.149) 0.786 |
| ≥6 (n=6623) | 0.762 (0.613, 0.948) 0.017 | 0.783 (0.691, 0.888) <0.001 | 0.868 (0.773, 0.974) 0.019 | 0.841 (0.749, 0.945) 0.005 | 0.871 (0.743, 1.022) 0.094 |
| *P* for trend | 0.007 | <0.001 | 0.005 | 0.001 | 0.034 |

DI-GM, dietary index for gut microbiota; OR, odds ratio; CI, confidence interval; BP, blood pressure; FBG, fasting blood glucose; HDL, high-density lipoprotein cholesterol; TG,triglyceride. Adjusted for age, gender, race, education level, marital status, PIR, BMI, Drinking status, Smoking status, eGFR, AST, ALT, TBil, UA, BUN, TC and LDH.

**Supplementary Table 5.** Analysis of the mediating role of albumin and SII in the association between DI-GM and metabolic syndrome.

| Characteristics | Mediation effect (95% CI), *P* value | | |  |
| --- | --- | --- | --- | --- |
|  | Total effect | Indirect effect | Direct effect | Mediation |
| Albumin | -0.0139(-0.0201,-0.0075) <0.001 | -0.001(-0.0015,-0.0006) <0.001 | -0.0129 (-0.0191, -0.0065) <0.001 | 7.44% |
| SII | -0.0139(-0.0202,-0.0076) <0.001 | -0.0003(-0.0005,-0.0001) 0.002 | -0.0137(-0.02,-0.0073)  <0.001 | 2.20% |

SII,systemic inflammation index; Adjusted for age, gender, race, education level, marital status, PIR, BMI, Drinking status, Smoking status, eGFR, AST, ALT, TBil, UA, BUN, TC and LDH.

**Supplementary Table 6.** The association between dietary components in DI-GM and MetS.

| Characteristics | Model 1 OR (95% CI) | Model 2 OR (95% CI) | Model 3 OR (95% CI) |
| --- | --- | --- | --- |
| Avocado | 0.998 (0.996, 0.999) 0.011 | 0.998 (0.997, 1.000) 0.03 | 0.999 (0.998, 1.000) 0.161 |
| Broccoli | 1.000 (0.999, 1.000) 0.199 | 1.000 (0.999, 1.000) 0.227 | 1.000 (0.999, 1.000) 0.388 |
| Chickpea | 0.993 (0.990, 0.997) 0.001 | 0.994 (0.991, 0.998) 0.005 | 0.997 (0.994, 1.000) 0.032 |
| Coffee | 1.000 (0.999, 1.000) 0.005 | 1.000 (0.999, 1.000) 0.029 | 1.000 (0.999, 1.000) 0.043 |
| Cranberry | 1.000 (0.999, 1.000) 0.068 | 1.000 (0.999, 1.000) 0.318 | 1.000 (0.999, 1.000) 0.208 |
| Fermented Dairy | 1.000 (1.000, 1.000) 0.022 | 1.000 (1.000, 1.000) 0.153 | 1.000 (1.000, 1.000) 0.124 |
| Fiber | 0.990 (0.986, 0.994) <0.001 | 0.988 (0.984, 0.992) <0.001 | 0.998 (0.994, 1.003) 0.547 |
| Green Tea | 1.000 (1.000, 1.000) <0.001 | 1.000 (1.000, 1.000) 0.001 | 1.000 (1.000, 1.000) 0.279 |
| Soybean | 1.000 (0.999, 1.000) 0.007 | 1.000 (0.999, 1.000) 0.011 | 1.000 (0.999, 1.000) 0.235 |
| Whole Grains | 0.972 (0.936, 1.009) 0.134 | 0.930 (0.890, 0.971) 0.001 | 0.993 (0.953, 1.035) 0.749 |
| Fat | 0.999 (0.998, 1.000) 0.187 | 1.001 (1.000, 1.002) 0.093 | 1.001 (0.999, 1.002) 0.32 |
| Refined Grains | 0.996 (0.985, 1.008) 0.531 | 1.026 (1.014, 1.038) <0.001 | 1.023 (1.011, 1.036) 0.001 |
| Processed Meat | 1.044 (1.022, 1.066) <0.001 | 1.069 (1.046, 1.092) <0.001 | 1.056 (1.031, 1.082) <0.001 |
| Red Meat | 1.027 (1.015, 1.038) <0.001 | 1.046 (1.033, 1.059) <0.001 | 1.027 (1.011, 1.044) 0.001 |

DI-GM, dietary index for gut microbiota; OR, odds ratio; CI, confidence interval.Model 1: Unadjusted for any covariates.Model 2: Adjusted for age, gender, and race. Model 3: Adjusted for age, gender, race, education level, marital status, PIR, BMI, Drinking status, Smoking status, eGFR, AST, ALT, TBil, UA, BUN, TC and LDH.

**Supplementary Table 7.** The association between DI-GM and MetS in the unweighted model.

| Characteristics | Model 1 OR (95% CI) | Model 2 OR (95% CI) | Model 3 OR (95% CI) |
| --- | --- | --- | --- |
| Continuous DI_GM (n = 23317) | 0.950 (0.933, 0.967) <0.001 | 0.910 (0.893, 0.927) <0.001 | 0.954 (0.934, 0.974) <0.001 |
| DI-GM group |  |  |  |
| 0–3 (n=5614) | 1[Ref] | 1[Ref] | 1[Ref] |
| 4 (n=5693) | 0.931 (0.858, 1.010) 0.086 | 0.908 (0.833, 0.989) 0.027 | 0.994 (0.904, 1.093) 0.9 |
| 5 (n=5387) | 0.880 (0.810, 0.956) 0.003 | 0.801 (0.734, 0.874) <0.001 | 0.882 (0.801, 0.972) 0.011 |
| ≥6 (n=6623) | 0.823 (0.760, 0.891) <0.001 | 0.689 (0.633, 0.750) <0.001 | 0.840 (0.765, 0.923) <0.001 |
| *P* for trend | <0.001 | <0.001 | <0.001 |

DI-GM, dietary index for gut microbiota; OR, odds ratio; CI, confidence interval. Model 1: Unadjusted for any covariates. Model 2: Adjusted for age, gender, and race. Model 3: Adjusted for age, gender, race, education level, marital status, PIR, BMI, Drinking status, Smoking status, eGFR, AST, ALT, TBil, UA, BUN, TC and LDH.

**Supplementary Table 8.** The association between DI-GM and MetS after multiple interpolation.

| Characteristics | Model 1 OR (95% CI) | Model 2 OR (95% CI) | Model 3 OR (95% CI) |
| --- | --- | --- | --- |
| Continuous DI_GM (n = 28273) | 0.937 (0.916, 0.957) <0.001 | 0.895 (0.875, 0.915) <0.001 | 0.954 (0.931, 0.976) 0.003 |
| DI-GM group |  |  |  |
| 0–3 (n= 6809) | 1[Ref] | 1[Ref] | 1[Ref] |
| 4 (n= 6981) | 0.882 (0.801, 0.971) 0.013 | 0.870 (0.785, 0.963) 0.009 | 0.945 (0.828, 1.080) 0.413 |
| 5 (n= 6520) | 0.827 (0.748, 0.914) <0.001 | 0.755 (0.681, 0.838) <0.001 | 0.873 (0.773, 0.986) 0.034 |
| ≥6 (n= 7963) | 0.761 (0.690, 0.839) <0.001 | 0.630 (0.570, 0.697) <0.001 | 0.812 (0.724, 0.912) 0.001 |
| *P* for trend | <0.001 | <0.001 | <0.001 |

DI-GM, dietary index for gut microbiota; OR, odds ratio; CI, confidence interval. Model 1: Unadjusted for any covariates. Model 2: Adjusted for age, gender, and race. Model 3: Adjusted for age, gender, race, education level, marital status, PIR, BMI, Drinking status, Smoking status, eGFR, AST, ALT, TBil, UA, BUN, TC and LDH.

**Supplementary Table 9.** The association between DI-GM and MetS after multiple interpolation.

| Characteristics | Model 1 OR  (95% CI) | Model 2 OR  (95% CI) | Model 3 OR  (95% CI) |
| --- | --- | --- | --- |
| Continuous DI_GM (n = 23317) | 0.933 (0.909, 0.957) <0.001 | 0.890 (0.867, 0.913) <0.001 | 0.915 (0.892, 0.939) <0.001 |
| DI-GM group |  |  |  |
| 0–3 (n=5614) | 1[Ref] | 1[Ref] | 1[Ref] |
| 4 (n=5693) | 0.890 (0.792, 0.999) 0.051 | 0.875 (0.773, 0.990) 0.037 | 0.894 (0.779, 1.025) 0.113 |
| 5 (n=5387) | 0.796 (0.714, 0.889) <0.001 | 0.716 (0.638, 0.804) <0.001 | 0.758 (0.670, 0.857) <0.001 |
| ≥6 (n=6623) | 0.751 (0.669, 0.843) <0.001 | 0.619 (0.552, 0.695) <0.001 | 0.689 (0.612, 0.776) <0.001 |
| *P* for trend | <0.001 | <0.001 | <0.001 |

DI-GM, dietary index for gut microbiota; OR, odds ratio; CI, confidence interval.Model 1: Unadjusted for any covariates.Model 2: Adjusted for age, gender, and race.Model 3: Adjusted for age, gender, race, education level, marital status, PIR, Drinking status, Smoking status, eGFR, AST, ALT, TBil, UA, BUN, TC and LDH.

**Supplementary Figure 1.** Variance Inflation Factor Values of Covariates in This Study.

**Supplementary Figure 2.** Average intake of DI-GM dietary components. Asterisks indicate statistically significant differences between the Without MetS group and the With MetS group. Values are presented to two decimal places. Significance levels: **p* < 0.05; ***p* < 0.01; ****p* < 0.001.
