# Supplementary figures and images for: Association Between the Dietary Index for Gut Microbiota and Metabolic Syndrome: Mediation Effects of Albumin and Systemic Immune‐Inflammation Index
Source: Food Sci Nutr. 2025 Nov 11;13(11):e71194. doi: 10.1002/fsn3.71194 (PMC12603787; doi:10.1002/fsn3.71194)

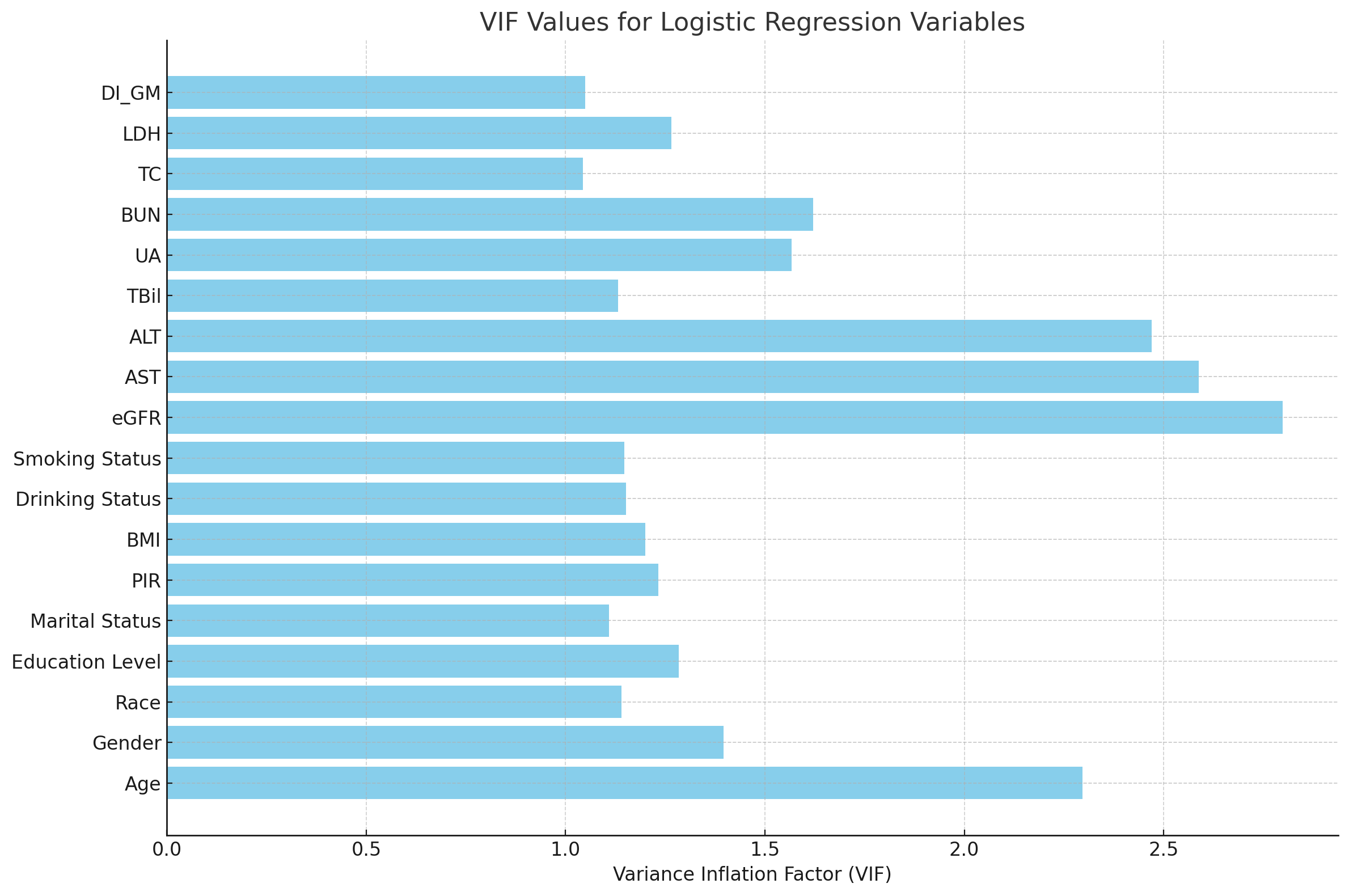

Supplement: Supplementary file 1 — Figure S1: Variance inflation factor values of covariates in this study. Figure S2: Average intake of DI‐GM dietary components. Table S1: Components and scoring criteria of DI‐GM. Table S2: Diagnostic criteria for metabolic syndrome. Table S3: Threshold effect analysis of DI‐GM and MetS associations based on a two‐segment linear regression model. Table S4: The association between DI‐GM and the individual components of MetS. Table S5: Analysis of the mediating role of albumin and SII in the association between DI‐GM and metabolic syndrome. Table S6: The association between dietary components in DI‐GM and MetS. Table S7: The association between DI‐GM and MetS in the unweighted model. Table S8: The association between DI‐GM and MetS after multiple interpolation. [file FSN3-13-e71194-s001.zip › fsn371194-sup-0001-FigureS1@Figure S1.png]

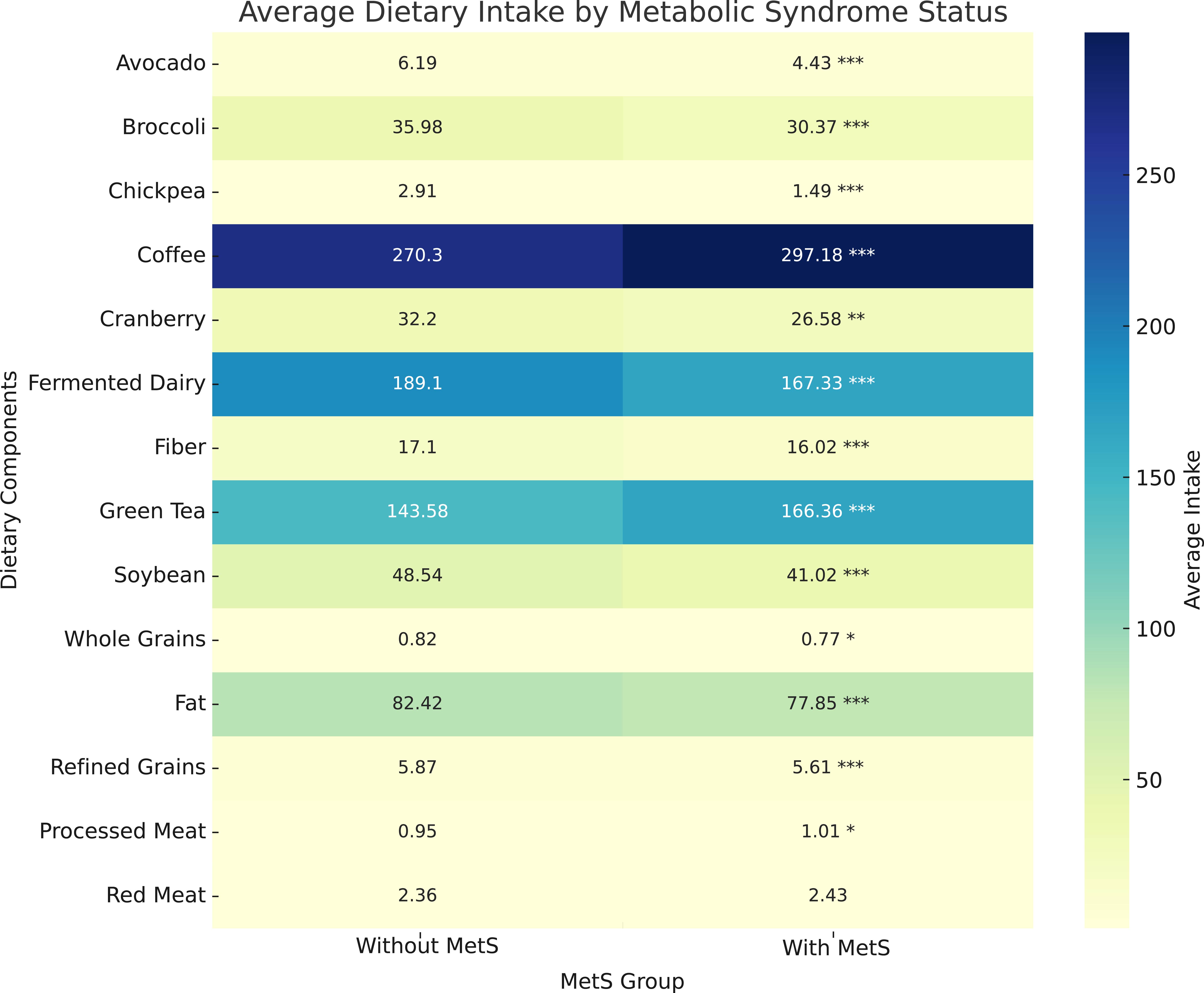

Supplement: Supplementary file 1 — Figure S1: Variance inflation factor values of covariates in this study. Figure S2: Average intake of DI‐GM dietary components. Table S1: Components and scoring criteria of DI‐GM. Table S2: Diagnostic criteria for metabolic syndrome. Table S3: Threshold effect analysis of DI‐GM and MetS associations based on a two‐segment linear regression model. Table S4: The association between DI‐GM and the individual components of MetS. Table S5: Analysis of the mediating role of albumin and SII in the association between DI‐GM and metabolic syndrome. Table S6: The association between dietary components in DI‐GM and MetS. Table S7: The association between DI‐GM and MetS in the unweighted model. Table S8: The association between DI‐GM and MetS after multiple interpolation. [file FSN3-13-e71194-s001.zip › fsn371194-sup-0002-FigureS2@Figure S2.jpg]
